# Safety, Efficacy and Evidence Base for Use of the Subcutaneous Implantable Cardioverter Defibrillator

**DOI:** 10.3390/jcm7030053

**Published:** 2018-03-11

**Authors:** Carmen Adduci, Francesca Palano, Pietro Francia

**Affiliations:** Division of Cardiology, Department of Clinical and Molecular Medicine, Sapienza University of Rome, St. Andrea Hospital, Via di Grottarossa 1035, 00189, Rome, Italy; carmen.adduci@uniroma1.it (C.A.); francesca.palano@uniroma1.it (F.P.)

**Keywords:** subcutaneous ICD, transvenous leads, sudden cardiac death

## Abstract

The trans-venous implantable cardioverter defibrillator (TV-ICD) is effective in treating life-threatening ventricular arrhythmia and reduces mortality in high-risk patients. However, there are significant short- and long-term complications that are associated with intravascular leads. These shortcomings are mostly relevant in young patients with long life expectancy and low risk of death from non-arrhythmic causes. Drawbacks of trans-venous leads recently led to the development of the entirely subcutaneous implantable cardioverter defibrillator (S-ICD). The S-ICD does not require vascular access or permanent intravascular defibrillation leads. Therefore, it is expected to overcome many complications associated with conventional ICDs. This review highlights data on safety and efficacy of the S-ICD and is envisioned to help in identifying the role of this device in clinical practice.

## 1. Introduction

The implantable cardioverter defibrillator (ICD) represents the only effective therapy to prevent sudden cardiac death (S-CD) in patients with structural heart disease or primary cardiac electrical syndromes on optimal medical therapy [1,2,3,4].

ICD technology evolved throughout years from devices that were only able to treat ventricular tachycardia or fibrillation (VT/VF) to modern systems that provide complex discrimination algorithms, bradycardia-pacing support and resynchronization therapy. While effective and relatively safe, transvenous ICDs (TV-ICDs) carry potential short- and long-term lead-related complications, including those that are associated with the implant technique (pneumothorax, venous thrombosis, hemopericardium, hemothorax) and those that are related to indwelling intracardiac leads (infections, lead failure) [3,5].

Noticeably, long-term complications are increasing with longer ICD patients’ follow-up. Indeed, it has been reported that lead failure occurs in up to 40% of transvenous leads eight years after implantation [6]. Failure occurs more frequently in young patients, who expose the leads to greater stress due to active life-style and longer life expectancy.

Moreover, long life expectancy is also associated with several pulse generator replacements and, therefore, to an increased risk of pocket infection with potential lead involvement and bloodstream dissemination [7]. Of note, failed and infected leads may need extraction, a complex procedure that is associated with morbidity and even mortality [7].

Recently, the unmet need for an ICD without endovascular leads led to the development of an entirely subcutaneous ICD (S-ICD).

## 2. The S-ICD System

The S-ICD consists of a subcutaneous pulse generator that is covered in a titanium case and a subcutaneous lead. The lead is composed of a proximal and a distal sensing electrode separated by a 3-inch shock coil. The S-ICD has not capability for bradycardia or anti-tachycardia pacing (ATP), but can deliver up to 30 s of post-shock transthoracic pacing [8]. The device has two programmable zones of tachycardia detection: a conditional VT zone and a VF zone. In the conditional zone, complex morphology-based algorithms discriminate VT/VF from supraventricular tachycardia (SVT), while in the VF zone heart rate is the only criterion to determine whether the DC shock will be delivered or not. 

## 3. S-ICD Implantation

The conventional S-ICD implantation is performed through three incisions: one on the left-lateral chest for the pulse generator pocket, and two parasternal incisions that allow for lead tunnelization. The first incision is over the fifth intercostal space between the mid and the anterior axillary lines, the second incision is parasternal just below xiphoid process and the third one is parasternal, at the level of the sternal notch. The pulse generator is positioned in a subcutaneous pocket created on the left lateral chest. The electrode is tunnelled from the parasternal incision through the pocket site, and then should lie parallel to the left side of the sternum, with its upper pole anchored at the level of the third incision.

S-ICD implantation is guided exclusively by anatomical landmarks, with the possibility to confirm electrode and pulse generator position by fluoroscopy.

The superior parasternal incision is more susceptible to infection, more likely to cause discomfort and may be aesthetically less acceptable than the other incisions. Therefore, a new implantation technique (the “two incisions technique”) has been developed that avoid the third superior parasternal incision by tunneling the defibrillation lead through a peel-away sheath introducer. The two-incision technique can be alternatively used and demonstrated similar safety and efficacy as compared to the three incision technique [9], and is currently the preferred implantation procedure. Recently, a shift from the subcutaneous to an intermuscular device implantation has been proposed and is rapidly gaining popularity. With the intermuscular approach, the S-ICD is placed in a virtual space between the external surface of the serratus anterior and the anterior surface of the latissimus dorsi muscles, offering the advantages of a better cosmetic outcome and the prevention of device damage during physical activities and skin erosion [10].

Using the two sensing electrodes and the generator itself as the third one, three sensing vectors are available to detect subcutaneous signals. The best vector is automatically selected by the system in order to avoid double QRS counting and T-wave oversensing.

**Figure 1:**
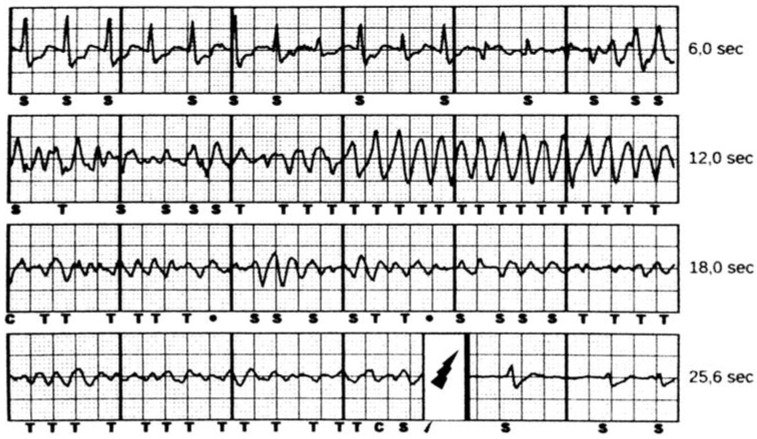
A 13 year old female patient with hypertrophic cardiomyopathy received an Subcutaneous implantable cardioverter defibrillator (ICD) (S-ICD) for primary prevention of sudden cardiac death (S-CD). The primary sensing vector was selected by the system and approved by the physician. One year after implantation, the patient experienced an episode of ventricular fibrillation. The figure shows the VF episode appropriately detected (T markers) and effectively treated by the S-ICD with an 80 J shock (lightning marker). Time to therapy was 16.5 s.

At the end of the procedure, VF is induced using 50 Hz current delivered by the device itself and the 65 J defibrillation threshold (DFT) is assessed. After implantation, the S-ICD is programmed to deliver only shocks at 80 J (Figure 1).

Unlike the TV-ICD, the defibrillation test is mandatory at present for S-ICD, as DFT may be more dependent on system positioning. Nonetheless, as growing data show that defibrillation failure rates with maximal energy output are comparable to the TV-ICD, the paradigm will likely shift away from DFT testing.

## 4. Screening and Eligibility

Pre-implant surface electrocardiogram (ECG) screening is needed to identify patients with unsuitable subcutaneous signals and a screening tool is used to confirm eligibility.

Three electrodes are positioned in the same chest position of the S-ICD lead distal and proximal sensing electrodes and the pulse generator. ECG recordings are collected in supine and standing postures and are superimposed on a plastic ruler provided with template boxes. Either the maximal R or S waves of the QRS complex have to fit between the horizontal solid and dashed lines in any template box. T-wave is required to fit within the boundaries of the template. A patient is considered suitable for S-ICD if all of the QRS-T complexes pass in any same lead supine and standing, at any gain, without changes of the R wave axis. In order to reduce subjectivity and increase efficiency, an automatic screening tool has recently been developed and equipped with a software that applies the same vector select algorithm and filters used by the S-ICD to sense the cardiac signals. 

## 5. Safety

Main data on S-ICD safety come from the pooled analysis of the U.S. IDE study, the EFFORTLESS registry [11], and from the midterm outcomes analysis of the EFFORTLESS [12]. The pooled analysis assessed 882 patients during a 651 ± 345 days follow-up [11], while in the extended follow-up of the EFFORTESS cohort, 985 patients were evaluated with a mean follow-up of 3.1 ± 1.5 years [12]. Moreover, recently published registries analysed the performances of S-ICD in the real world setting and evaluated in-hospital and short-term outcomes among thousands of S-ICD recipients [13,14].

### 5.1. Infections

The most common complication associated with S-ICD implants is device infection. The pooled analysis of the IDE and EFFORTLESS studies reported a 2% device infection, with 1.7% requiring explant/revision [11]. When complications were analysed according to the enrolment period, a decreasing incidence of infections with increasing operator experience has been observed [15], which is consistent with a “learning curve”. The resulting reported global infection rate (2.4% over two years) was comparable to that of TV-ICDs [16,17] (Table 1 and Table 2).

The results of the midterm analysis of the EFFORTLESS registry are similar: 2.9% of patients had infection, with 2.4% requiring device explant during a 3.1 years average follow-up [12]. All prosthesis infections have the potential to become systemic. Indeed, TV-ICD infections are associated with a 22% to 54% risk of bacteraemia and endocarditis [20,21]. Instead, none of the S-ICD infections that are reported in the IDE and EFFORTLESS studies resulted in systemic dissemination [25,26]. Trans-venous device infections are unlikely to resolve with antibiotic therapy, and lead extraction if often required. Common complications of lead extraction include hemopericardium, cardiac tamponade, pneumothorax, stroke, and vascular damage. Of note, the overall complication rate in patients undergoing trans-venous lead extraction is higher than previously reported, with mortality rates exceeding 2% [27].

### 5.2. Implant Site Complications

Implant-site hematoma and device erosion are less common complications. The incidence of hematoma was consistently less than 1% across different registries [11,12,13,14] (Table 3). Instead, higher hematoma rates have been reported for TV-ICDs, ranging from 0.86% to 2.4% [5,7,22].

Device erosion was reported during the early experience, possibly being due to the large first generation S-ICD and to the implant site in the axilla region. Current rates of erosion are in the range of 1.2% to 1.8% [11,12] and are consistently decreasing with greater operators experience [15], reduced dimensions of the new device, and use of the intermuscular implantation technique.

### 5.3. Lead/Pulse Generator Related Complications

Suboptimal system position was reported in less than 2% of patients, and lead migration is a rare complication (<1% of patients) [11,12] (Table 3). Of note, even when considering long follow-up studies [12,28], there are no reported lead malfunctions or failures worldwide. However, as lead failures typically develop many years after implant, longer-term follow-up is needed to ascertain the true incidence of S-ICD lead failure. 

### 5.4. Inappropriate Shocks

In the first S-ICD trials, inappropriate shock rate was reported as 5% to 25%, and was mainly due to supraventricular tachycardia (SVT), T-wave oversensing and lead migration. Careful patient selection and pre-implantation ECG screening is probably the most effective way to avoid inappropriate shocks [29]. Exercise testing during pre-implant ECG screening has been suggested [30] and may be particularly useful in assessing vector eligibility in patients with hypertrophic cardiomyopathy [31,32]. Moreover, post-implant exercise may be used to acquire a new template and to improve discrimination when heart rate-dependent changes in QRS morphology occur [30], or to optimize vector selection in patients with specific diseases [33].

Combining careful pre-implant screening and optimized vector selection with enhanced device programming led to a further reduction in inappropriate S-ICD interventions. Indeed, programming and discrimination algorithms evolved significantly since the introduction of the S-ICD. Initially, S-ICDs were programmed with a single 180 bpm shock zone, leading to a high rate of interventions for sinus tachycardia and other SVTs.

The START (Subcutaneous versus Transvenous Arrhythmia Recognition Testing) trial [8], which compared discrimination algorithms of S-ICDs and TV-ICDs, reported that adding a conditional VT zone (dual zone programming) resulted in a 98% specificity for appropriate detection of SVTs for the S-ICD.

Accordingly, dual zone programming (a 170–220 bpm zone with SVTs discrimination algorithms plus a zone for heart rate >220 beats/min) significantly decreased the rate of inappropriate shocks [15].

Finally, the introduction of advanced discrimination algorithms and ad-hoc filters that were designed to avoid T-wave oversensing further decreased the rate of inappropriate shocks [34].

The IDE and EFFORTLESS pooled analysis reported an incidence of inappropriate shock at 3 years of 11.7%, due to SVT (24%) or T-wave oversensing (39%) [11]; similar rates were reported in the midterm outcomes of the EFFORTLESS registry [12].

## 6. Efficacy

### 6.1. Acute Defibrillation Test

At S-ICD implantion, defibrillation threshold testing (DFT) is typically performed to assess proper device implantation (i.e., appropriate sensing of ventricular arrhythmia and shock efficacy). The European clinical trial reported 100% sensitivity for detection of induced VF and 98% shock efficacy [35]. The midterm analysis of the EFFORTLESS registry [12] reported a failure conversion rate of 0.2%. DFT was ≤65 J in 91.6% of patients and 70 to 80 J in 4.4% (3.5% unrecorded energy) [12].

In the S-ICD post approval study [13], DFT testing was performed in the 86.3% of the patients, with a failure conversion rate of 0.4%. First successful conversion was achieved in 98.7% of the patients (91.2% ≤65 J and 7.5% 70 to 80 J). In the US S-ICD Trends [14], DFT testing was performed in 75% of the patients, reporting a similar high efficacy (92.7% ≤65 J and 7% 70 to 80 J).

### 6.2. Spontaneous VT/VF Events

In early longitudinal studies, the S-ICD efficacy in converting spontaneous ventricular arrhythmia was in the range of 95.2% to 100% [26,35,36].

In the midterm analysis of the EFFORTLESS (994 patients), after a follow-up of 3.1 ± 1.5 years, a total of 104 patients (10.6%; annual incidence 3.4%) had 278 appropriately treated VT or VF episodes, including 86 storm episodes. Of non-storm VT or VF episodes, 88.5% were converted with a single shock and 97.4% (187 of 192 episodes) within five shocks available. All of the patients survived their arrhythmic events. First shock conversion effectiveness was 90.5% for appropriately treated discrete episodes of monomorphic ventricular tachycardia (MVT) and 86.6% for polymorphic ventricular tachycardia (PVT) or VF. Among 13 VT/VF storms, (86 episodes) 12 were successfully converted. When considering overall shock efficacy, the conversion failure rate was 2.6%. Similar conversion failure rate was reported for TV-ICDs (2.6% in the SIMPLE trial [37], 1.6% in the NORDIC [38], and 1.6% in the SCD-HeFT [4]).

## 7. Patient Selection

The S-ICD was approved for the prevention of sudden cardiac death among candidates to an ICD without indication for anti-bradycardia pacing or CRT, recurrent monomorphic VTs responsive to ATP, or pre-existing unipolar pacemaker leads. Patients enrolled in early S-ICD prospective registries were relatively young and with less advanced heart disease [25]. Often, high risk of infections, congenital heart disease, and poor vascular access were strong determinants of device selection. However, contemporary S-ICD patients are more severely diseased, older, and have more comorbidities [13,14]. Indeed, in the S-ICD Post-Approval Study [13], the mean age was 53 ±15 years, 74% of the patients had heart failure, 34% had diabetes, and 26% had chronic kidney disease. Clinical characteristics of patients that were enrolled in the recently published US-S-ICD Trends study [14] are similar, patients with previous myocardial infarction (45%) and low ejection fraction (average 32%) being largely represented (Table 2). These real-life data are consistent with a shift towards use of S-ICD in contemporary ICD candidates.

Young patients with cardiomyopathies or channelopathies are ideal candidates for the S-ICD. Indeed, lead-related complications of the transvenous ICD have been reported to be as high as 2–4% per year in these patients [39,40]. Intravascular lead failure and infection are of outmost importance in patients with long life-expectancy and low risk of dying from non-arrhythmic causes, who are those more exposed to long-term complications. Moreover, in patients with inherited primary electrical diseases (e.g., long and short QT syndrome, catecholaminergic polymorphic VT, Brugada syndrome, idiopathic VF) the clinical arrhythmia are usually VF or polymorphic VT, which are unresponsive to ATP. In this regard, the S-ICD was shown to be safe and effective in cardiomyopathy patients [41,42]. Moreover, because of the small risk of systemic infection [25] and anticipated increased durability of the lead [23], the S-ICD is an appealing alternative to the TV-ICD in most cardiomyopathy patients without a pacing indication. As far as Brugada syndrome is concerned, polymorphic VT or VF have been consistently shown to be the most common arrhythmia in these patients. Recently, monomorphic VTs (MVTs) have also been reported in Brugada syndrome patients [43]. However, it is still unclear whether MVTs in Brugada patients are mechanistically similar to polymorphic VTs, a distinct disease-related entity, the result of a misclassification bias, or an epiphenomenon [44]. Moreover, while the hypothesis of MVTs as a distinct disease-related entity is intriguing, it is unclear whether anti-tachycardia pacing may be effective in this specific setting and would balance the long-term risk of lead-related complications.

## 8. Conclusions and Perspectives

After almost three decades of innovation in ICD technology, the introduction of an implantable defibrillator that is radically different in terms of surgical implantation and arrhythmia detection/discrimination represents a technical and clinical breakpoint in the long fight against sudden cardiac death. Data from clinical studies support S-ICD efficacy and safety in detecting and terminating ventricular arrhythmia. While in the early S-ICD experience the ideal candidates were relatively young and with less advanced heart disease, the paradigm is rapidly shifting towards use of the S-ICD in contemporary ICD candidates. Indeed, according to current guidelines [45], all ICD candidates have a class IIa indication for the implantation of an S-ICD unless pacing for bradycardia, anti-tachycardia pacing, or resynchronization therapy is needed or anticipated. Of note, patients who meet criteria for an ICD who have inadequate vascular access or are at high risk for infection (including those with previous TV-ICD infection), and in whom pacing is neither needed nor anticipated, have a class I indication [45]. As the question whether an S-ICD may be considered completely equivalent to a TV-ICD in patients without pacing indication is presently unaddressed, the PRAETORIAN trial [46] is currently randomizing patients to an S-ICD or a TV-ICD and will compare the two devices in terms of safety and efficacy.

## Figures and Tables

**Table 1:** S-ICD recipients in early as compared with contemporary real-life registries.

	EFFORTLESS	S-ICD Post Approval Study	US S-ICD Trends
N	985	1637	3717
Males	72%	69%	69%
Age (years)	48 ± 17	53 ± 15	53 ± 15
CAD (previous MI)	29%	33%	40%
EF (mean)	43 ± 18	32 ± 14	32 ± 14
Hypertrophic Cardiomiopathy	11%	NA	5%
Channelopathies	20%	4%	8%
Diabetes	11%	34%	38.5%
Atrial Fibrillation	16%	16%	20%
CKD	8%	26% (dyalisis 13%)	41% (dyalisis 20%)

CAD: Coronary artery disease; MI: myocardial infarction; EF: ejection fraction; CKD: Chronic kidney disease.

**Table 2:** Comparison of S-ICD and TV-ICD complications.

Complications	SICD	TV-ICD	Reference
**Infections**			
Infection rate (per year)	2%	1.6%	[16,17,18]
need for explant	1.7%	>50%	[19]
endocarditis/bacteraemia	0%	22–54%	[20,21]
**Implant Site Complications**			
Haematoma	4%	0.86–2.4%	[5,7,22]
Device erosion	1.2–3%	1.5%	[12,23]
**Lead or Pulse Generator Complications**			
Inappropriate shocks (per year)	1.6%	7–10% (first year)	[12,24]
		18% (5 year follow-up)	
Electrode dislodgement	0.6%	1.8% (single/dual ICD) 5.9% (CRT)	[11,12,24]

**Table 3:** S-ICD complications across prospective studies and real-life registries.

	Pooled Analysis IDE + EFFORTLESS	EFFORTLESS Midterm	S-ICD Post Approval Study	US S-ICD Trends
Infection requiring removal/revision	1.7%	2.4%	1.2%	0.05%
Erosion	1.2%	1.7%		
Hematoma	0.4%	0.9%	0.4%	0.3%
Discomfort	0.9%	0.8%	0.1%	
Lead dislodgment	0.6%	0.7%		0.1%
Superficial Infection	0.3%	0.5%	0.1%	
Suboptimal PG or/and lead position	1.4%	1.6%	0.5%	
Inappropriate shocks: oversensing	4.6%	5.1%	0.2%	
Inappropriate shocks: SVTs	2.8%	2.3%		
Total complications	9.6%	11.7%		

PG: pulse generator, SVT: supraventricular tachycardia.
